# Management of type I gastric-neuroendocrine tumors: the less the better?

**DOI:** 10.3389/fendo.2025.1666699

**Published:** 2025-09-01

**Authors:** Roberta Elisa Rossi, Benedetta Masoni, Valeria Poletti, Roberta Maselli, Silvia Carrara, Alexia Francesca Bertuzzi, Silvia Uccella, Andrea Gerardo Antonio Lania, Alessandro Zerbi, Cesare Hassan, Alessandro Repici

**Affiliations:** ^1^ Gastroenterology and Endoscopy Unit, IRCCS Humanitas Research Hospital, Milan, Italy; ^2^ Department of Biomedical Sciences, Humanitas University, Milan, Italy; ^3^ Hematology and Oncology, IRCCS Humanitas Research Hospital, Milan, Italy; ^4^ Pathology Service, IRCCS, Humanitas Research Hospital, Milan, Italy; ^5^ Endocrinology and Diabetology Unit, IRCCS Humanitas Research Hospital, Milan, Italy; ^6^ Pancreatic Surgery Unit, IRCCS Humanitas Research Hospital, Milan, Italy

**Keywords:** gastric neuroendocrine tumors, gastric carcinoids, autoimmune chronic atrophic gastritis, endoscopic resection, recurrence, survival

## Abstract

**Background and aims:**

Type I gastric neuroendocrine tumors (gNETs) are known for their favorable prognosis. We aimed to present a real-life experience at a tertiary referral center.

**Materials and methods:**

Retrospective analysis of patients diagnosed with type I gNETs at our Institution between 2014 and 2024.

**Results:**

A total of 36 lesions were identified in 23 patients, with a median tumor size of 7 mm (range 2-20 mm). There were 29 out of 36 lesions that were G1, and 7 were G2. In 13 cases, endoscopic ultrasound (EUS) was performed prior to resection, revealing lymph node involvement in one 20-mm G1 lesion that required surgery. A 15-mm G2 lesion underwent surgery. In the remaining 34 lesions, endoscopic resection was performed: forceps in 5, cold-snare polypectomy in 4, hot-EMR in 22, EMR-cap in 1, ESD in 1, hybrid-ESD in 1. Among those, one 5-mm G2 lesion, previously removed via simple polypectomy, required surgery due to the 14.5% Ki-67 index. The median follow-up was 14 months (range 1-120), with 10 cases of local recurrence in 6 patients, median tumor size 3 mm (range 2-8 mm), all G1. In three cases, endoscopic surveillance was indicated; seven NETs underwent endoscopic resection (three forceps, two EMR-cap, two EMR), with EUS being performed in four cases with negative results. No local/distant metastases nor tumor-related deaths occurred.

**Conclusions:**

Present data confirm an indolent behavior for type I gNETs. Preoperative EUS staging led to a change in the management in one case, which highlights the need of dedicated studies to identify predictive factors to stratify risk and plan the management of these neoplasms.

## Introduction

The incidence of neuroendocrine tumors (NETs) arising in the gastrointestinal tract is steadily increasing due to the widespread use of endoscopy, reaching approximately 2.5-5 cases per 100,000 population per year.

Gastric NETs (gNETs) account for 5%-15% of all gastrointestinal (GI) NETs (1, 2) and 3% of all gastric tumors (1, 2). They are often diagnosed incidentally during upper GI endoscopy, as they are usually non-functioning tumors. Gastric NETs can be divided into three major categories, with different behavior and prognosis. Type I gNETs are associated with chronic atrophic gastritis (CAG) and represent 70%-80% of all gNETs: serum gastrin levels rise in response to gastric achlorhydria and stimulate enterochromaffin-like (ECL) cell hyperplasia. Type II gNETs (5% of all gNETs) occur due to hypergastrinemia associated with Zollinger–Ellison syndrome (ZES) and multiple endocrine neoplasia type I (MEN-I) syndrome. Type III gNETs (15%–25% of all gNETs) are sporadic lesions not associated with hypergastrinemia (3).

Due to their etiopathogenesis, type I gNETs may present as multifocal. They are usually diagnosed in adult patients with a mean age of 60-70 years and are more common in women due to the underlying autoimmune disease. Long-term survival does not differ from that of the general population because type I gNETs are usually indolent, well-differentiated, grade 1 (G1, Ki-67 <3%) tumors. Their risk of metastasis is only 0%-2% (4). Cumulative survival for patients with a history of type I gNETs is close to 100% at 5 years (5).

Endoscopically, they appear as multiple polypoid lesions less than 1 cm in size (6, 7). According to available guidelines (3), endoscopic ultrasound (EUS) should be performed in case of lesions >1 cm or smaller G2 grade lesions, prior to any resection, to determine the depth of local invasion and to assess local lymph node involvement. Radiological imaging (computed tomography (CT) scan, magnetic resonance imaging (MRI), and somatostatin receptor 68Ga positron emission tomography (PET/CT)) is generally not required for type I gNETs, except in the presence of lymph node metastases on EUS or in the presence of high-risk features (high G2, vascular invasion, muscularis propria invasion) (3).

Endoscopic resection appears to be an effective therapeutic option for patients with type I gNETs that are smaller than 2 cm and confined to the mucosal and submucosal layers. Advanced endoscopic techniques including endoscopic mucosal resection (EMR) or endoscopic submucosal dissection (ESD) are generally recommended. In fact, en-bloc resection rates are greater than 90% for EMR and 95% for ESD, with local recurrence rates at the 5-year follow-up of 6.5% and 2.4% for EMR and ESD, respectively (7, 8). Surgical excision is rarely necessary for type I gNETs and might be considered only in selected cases, i.e., tumor size larger than 2 cm, increased depth of invasion, poor histology, local positive lymph nodes, or lack of expertise in performing advanced endoscopic procedures (9, 10). Medical therapy with somatostatin analogues (SSAs) has been suggested, although it remains controversial, in cases of multifocal disease (11, 12).

After resection, lifelong endoscopic surveillance every 6 to 12 months is recommended to detect early development of other gNETs associated with persistent hypergastrinemia.

Over the past decade, there have been some updates to the dedicated European guidelines (3) for the management of these malignancies, and there is increasing debate to support a possible more conservative management of these tumors (13).

Based on these observations, we aimed to present a real-life clinical experience in a tertiary referral center for both neuroendocrine tumors and endoscopy to better understand the proper management of these neoplasms.

## Materials and methods

This is a retrospective case series. We collected data on all consecutive patients with a gastric resected lesion, which was histologically diagnosed as a type I gNET at the IRRCS Humanitas Research Hospital, Rozzano, Milan, Italy, between 2014 and 2024.

Exclusion criteria included age <18 years and lack of pertinent clinical/endoscopic information. Informed consent was waived due to the use of retrospective historical de-identified data.

Data on patients’ general characteristics, endoscopic lesion features [including tumor location, size, and morphology according to the Paris classification (14)], EUS results whenever available, histology, resection technique, complications, outcomes, and follow-up modalities were collected from electronic medical records and revision of procedure images. Resection techniques were analyzed in relation to the size, depth, and grade of the tumor.

The diagnosis was based on the recognition of well-differentiated neuroendocrine morphology, supported by the expression of general neuroendocrine markers (chromogranin A and synaptophysin). The subtyping was performed according to the features of the surrounding non-neoplastic mucosa, in which an atrophic gastritis with the morphological features of autoimmune gastritis was found. Tumors were graded gas G1, G2, or G3 lesions according to the WHO classification based on the tumor mitotic count and Ki-67 proliferation index (15). The depth of the tumor invasion was assessed by EUS. The endoscopic resection techniques included:

- Biopsy forceps or cold snare polypectomy;
- Endoscopic mucosal resection (EMR) with hot snare, preceded by submucosal infiltration with methylene blue, adrenaline, and saline solution;
- Cap-assisted EMR (c-EMR) with hot snare, performed after submucosal infiltration and the use of a transparent plastic cap positioned at the distal end of the endoscope to enhance lesion lifting by suction;
- Endoscopic submucosal dissection (ESD) using a dedicated through-the-scope needle-type knife, preceded by submucosal infiltration.

Surgical resection included gastric wedge resection, partial gastrectomy, or total gastrectomy, with or without regional linfo-adenectomy.

For all the patients, follow-up and recurrence data were reported.

All the cases were discussed at the multidisciplinary NET meeting at our European Neuroendocrine Tumor Society (ENETS) excellence center.

## Results

Between 2014 and 2024, a total of 36 lesions were identified in 23 different patients, 8 men (34.7%) and 15 women (65.3%). The mean age at the diagnosis was 60.7 years (SD 12.4).

The majority of the lesions were localized in the gastric body (25 NETs, 69.4%), whereas 8 were found in the fundus (22.2%) and 3 in the antrum (8.3%). Macroscopically, according to the endoscopic Paris classification, the lesions were characterized as follows: 26 were sessile (Is, 72.2%), 5 were slightly elevated flat lesions (IIa, 13.8%), and 5 slightly elevated flat lesions with central depression (IIa+ IIc). The median diameter was 7 mm (range 2-20 mm).

In 13 cases (36.1%), a preoperative staging EUS was performed before resection. According to EUS findings, only one case (a 20 mm, G1 lesion) exhibited evidence of lymph node involvement that subsequently required surgical resection. In another case (15 mm, G2 lesion), surgery with video laparoscopic gastric wedge resection was performed, whereas 34 lesion underwent endoscopic resection. In details, the endoscopic resective technique included forceps in 5 cases, cold snare polypectomy in 4, hot-EMR in 22, EMR-cap in 1, ESD in 1, and hybrid ESD in 1. Among those, one 5-mm G2 lesion, which had initially undergone resection via cold snare polypectomy, later underwent radicalization with surgical wedge resection due to the finding of a high Ki-67 index on the resected specimen (ki-67 14.5%) and to the evidence of incomplete resection (R1). Out of the 36 lesions, 29 were G1, whereas 7 were G2 (19.4%). No perioperative complications were reported.

**Figure 1:**
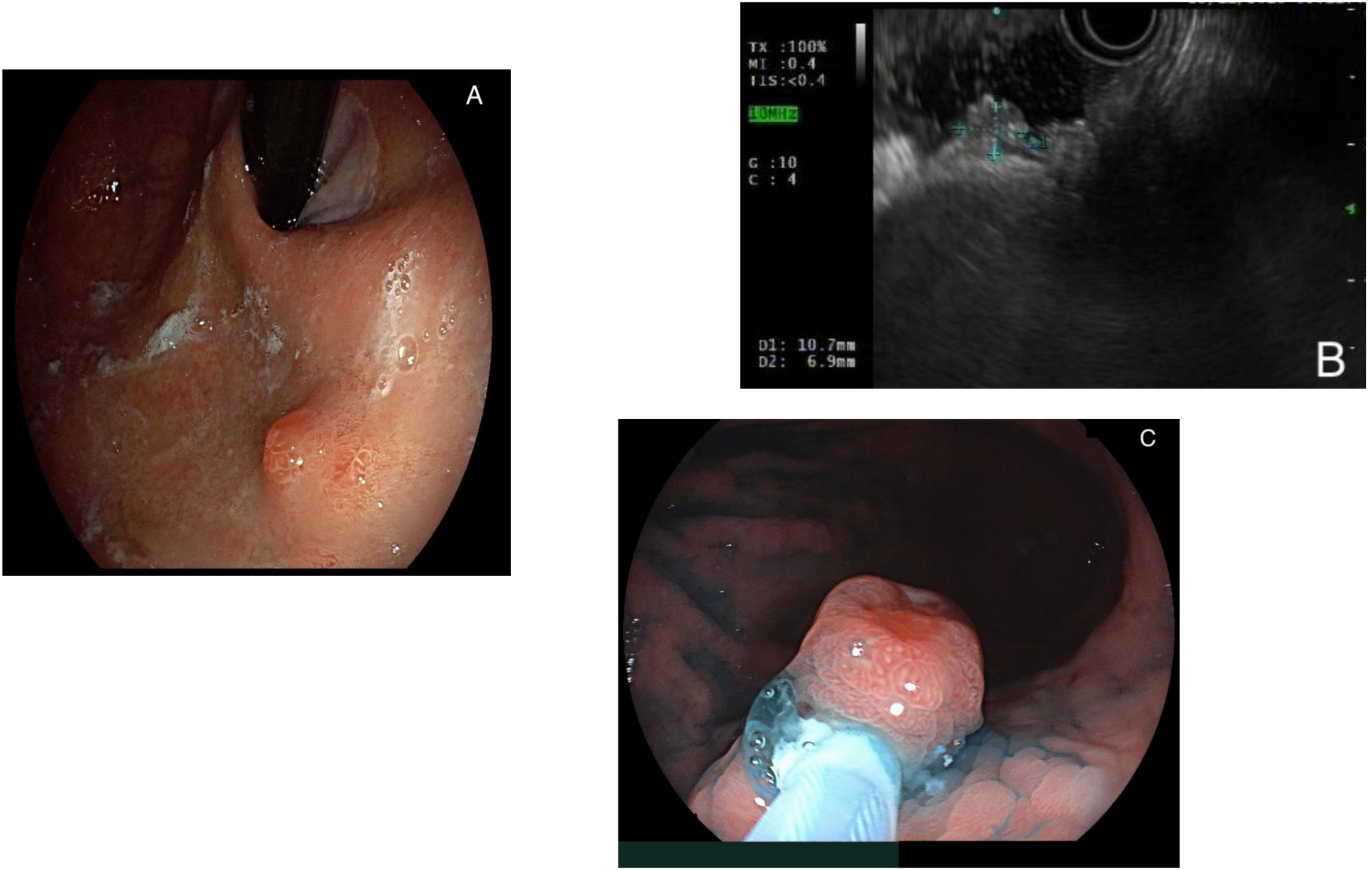
**(A)** Endoscopic appearance of a gastric neuroendocrine tumor (gNET), visualized in retroversion; **(B)** endoscopic ultrasound aspect of a gNET; **(C)** endoscopic resection via hot- endoscopic mucosal resection (EMR) of a gNET.

**Figure 1** represents the endoscopic appearance of a gNET.

In nine cases (25%), the state of the margins was assessable, and in only in two cases they were positive for the presence of neoplastic cells (R1 resection). One 15-mm G2 lesion had been removed via hot-EMR and later developed recurrence, and one 5-mm G2 lesion later underwent surgical radicalization as previously described.

**Table 1** summarizes the characteristics of these 36 lesions.

**Table 1:** Characteristics of the 36 newly diagnosed gastric neuroendocrine tumors (gNETs).

Sex	Age at diagnosis (years)	Dimension (mm)	EUS before resection	Resective technique	Grading	Ki-67 index (%)	Margins	Recurrence
M	51	20	Yes *	Surgical resection	G1	0.5	NA	No
F	79	7	Yes	Hot EMR	G2	4	R0	No
M	79	15	Yes	Surgical resection	G1	2	R0	Yes
M	41	15	Yes	EMR-cap	G1	1	R0	No
F	55	7	No	Hot EMR	G2	10	R0	No
M	79	15	Yes	Hot EMR*	G2	4	R1	Yes
F	60	8	Yes	Hot EMR	G1	1	NA	Yes
F	62	5	Yes	Hot EMR	G1	<2	NA	No
M	49	10	No	Hot EMR	G2	18	NA	No
F	55	3	No	Cold snare polipectomy	G1	2	NA	No
F	52	7	No	Hot EMR	G1	<2	NA	Yes
M	65	14	No	ESD	G1	1	R0	Yes
F	41	6	No	Cold snare polipectomy	G1	1	NA	No
F	41	8	Yes	Hot EMR	G2	3	NA	Yes
F	68	2	No	Forceps	G1	1	NA	No
F	71	7	No	Forceps	G1	2	NA	No
F	73	10	No	Hot EMR	G1	1	R0	No
F	64	5	No	Cold snare polipectomy+ surgical radicalization	G2	14.5	R1	No
F	60	10	Yes	Hot EMR	G1	2	NA	No
F	60	8	Yes	Hot EMR	G1	2	NA	No
F	79	6	No	Hot EMR	G1	1	NA	No
F	79	4	No	Hot EMR	G1	1	NA	No
F	79	6	No	Hot EMR	G1	1	NA	No
F	79	4	No	Hot EMR	G1	1	NA	No
F	79	9	No	Hot EMR	G1	1	NA	No
F	79	5	No	Hot EMR	G1	1	NA	No
F	79	4	No	Hot EMR	G1	1	NA	No
F	79	4	No	Hot EMR	G1	1	NA	No
M	59	9	No	Hot EMR	G1	2	NA	No
M	59	9	No	Hot EMR	G1	2	NA	No
M	59	11	No	Hot EMR	G1	2	NA	No
F	69	2	Yes	Forceps	G1	1	NA	No
F	69	2	Yes	Forceps	G1	1	NA	No
F	69	3	Yes	Forceps	G1	1	NA	No
F	56	5	No	Hybrid ESD	G2	5	R0	No
M	51	4	No	Cold snare polipectomy	G1	1	NA	No

M, male; F, female.

*: presence of lymph node involvement at EUS evaluation.

EUS, endoscopic ultrasound.

EMR, endoscopic mucosal resection.

ESD, endoscopic submucosal dissection.

NA, not available.

The follow-up period was defined as the interval between the date of NET resection and the last outpatient endoscopic evaluation. All patients underwent a follow-up endoscopic examination 6 months after resection and subsequent visit to dedicated outpatient clinic at which the timing of the next endoscopic follow-up was established.

The median follow-up period in our cohort was 14 months (range 1-120), during which 10 cases of local recurrence were reported (55.5%) in 6 patients (3 women, 50%). Among those patients, the characteristics of the initially diagnosed lesion that recurred following resection were as follows: a median size of 7.5 mm (range 2-14 mm), 5/6 (83.3%) G1 lesions and 1/6 (16.7%) G2 lesion, and 33.3% were multifocal at the time of diagnosis. The median time interval between resection and first recurrence was 31 months (range 11-57).

Among the 10 cases, in 3 cases endoscopic surveillance was indicated, whereas 7 NETs underwent endoscopic resection as follows: forceps in 3 cases, EMR-cap in 2, hot EMR in 2. In this context, preoperative EUS was performed in 4 cases (57.1%) with negative results. In 100% of cases, the NETs were identified as G1.

No local and/or distant metastases nor tumor-related deaths occurred.

The characteristics of these 10 recurrent lesions are summarized in **Table 2**.

**Table 2:** Characteristics of the seven recurrences treated endoscopically.

Sex	Recurrence time (months)	Age at recurrence (years)	Dimension (mm)	EUS before resection	Resective technique	Grading	Ki-67 index (%)	Recurrence
M	16	81	2	No	Forceps	G1	1	No
F	27	64	8	Yes	Hot EMR	G1	2	Yes
F	53	67	3	No	Forceps	G1	1	No
F	33	43	7	Yes	EMR-cap	G1	2	Yes
F	33	43	6	Yes	EMR-cap	G1	2	Yes
F	33	43	3	No	Forceps	G1	2	Yes
F	34	46	3	Yes	Hot EMR	G1	1	No

M, male; F, female.

EUS, endoscopic ultrasound.

EMR, endoscopic mucosal resection.

**Figure 2:**
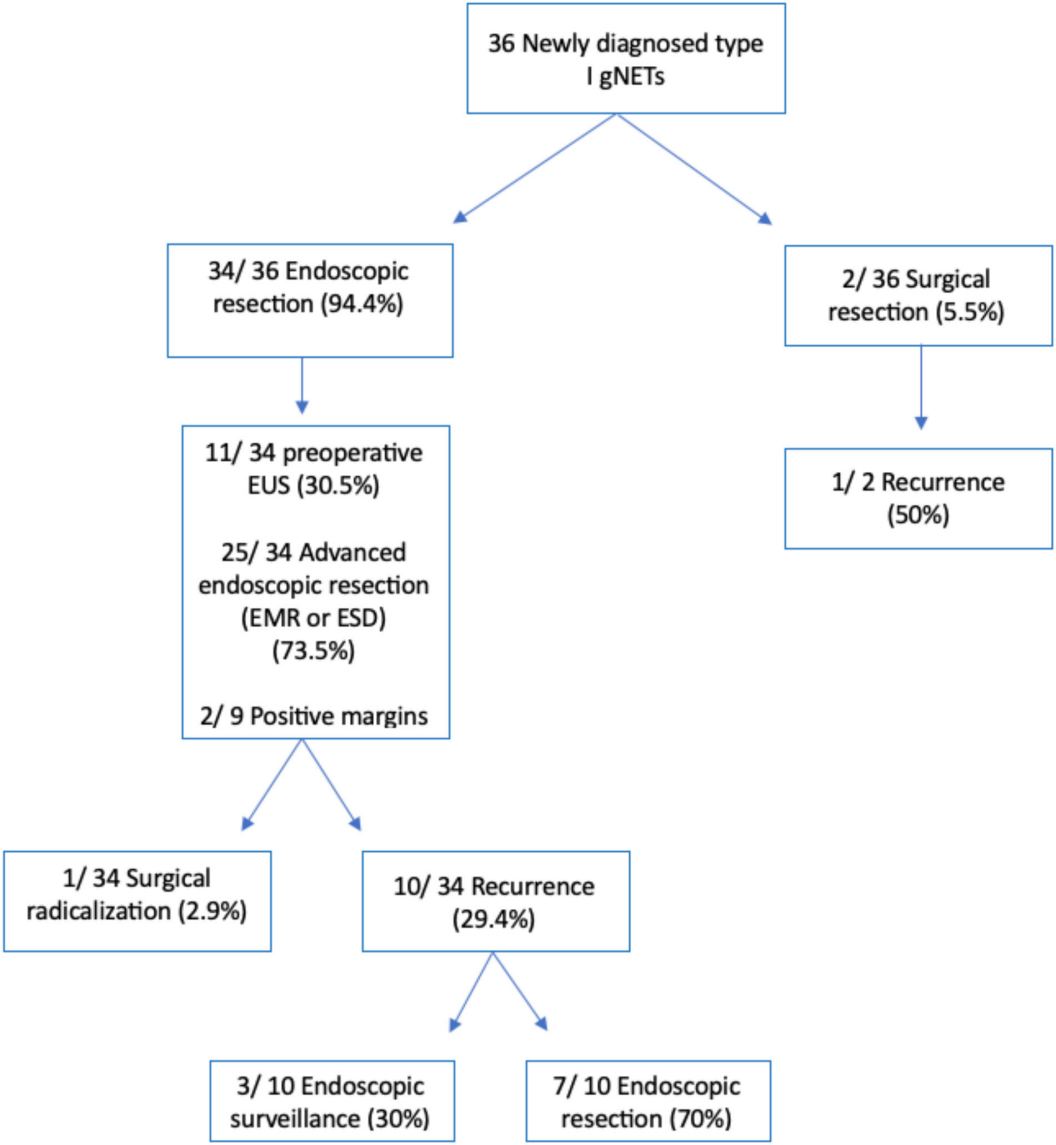
Flowchart depicting the methods used for the resection of gastric neuroendocrine tumors from our series and the resulting outcomes. *EMR, endoscopic mucosal resection; ESD, endoscopic submucosal dissection.

A flowchart summarizing the methods used for the resection of gNETs from our series and the resulting outcomes is depicted in **Figure 2**.

## Discussion

According to the present series, in line with current literature (3), type I gNETs are characterized by an indolent behavior, as neither the fact that some gNETs were resected with a simple endoscopic technique nor the significant percentage of recurrence had impact on overall survival.

In recent years, there have been some developments in the management of gNETs, particularly with regard to preoperative staging and the resective technique itself (3). While the Ki-67 proliferation index [and the consequent grading according to the WHO classification, (16)] is generally considered to play a significant prognostic role for digestive NETs, its prognostic role in type I gNETs is less established. The main prognostic factor determinant for the development of metastatic disease in this context appears to be the tumor size with a threshold of 10 mm, as previously established (17, 18).

At the present time, treatment options for type I gNETs include surveillance, endoscopic resection, SSAs, and surgery in limited cases only (3). In accordance with the current guidelines, endoscopic resection is recommended for G1-G2 lesions exceeding 10 mm in size in the absence of invasion of the muscle layer or lymph node involvement at the preoperative EUS evaluation.

By contrast, surgical resection is limited to selected cases: (1) lesion larger than 20 mm; (2) lesions <20 mm with high-risk features on biopsies (e.g., high Ki-67 index); (3) lesions of any size with evidence of muscle layer invasion or lymph node involvement at EUS or axial imaging assessment (3).

In line with available evidence, in our series, 34 lesions were resected through endoscopic techniques, of which 9 lesions measured at least 10 mm (26.4%) and 10 measured between 7 and 9 mm (27.7%). Two patients underwent upfront surgical resection: one case of 20-mm G1 lesion with evidence of lymph node involvement at EUS evaluation, and another case of a 15-mm flat G2 lesion with central depression (IIa+ IIc). Lastly, one 5-mm G2 lesion, previously endoscopically resected, underwent radicalization with surgical wedge resection due the finding of a high Ki-67 index on the resected specimen (i.e., 14.5%).

With regard to smaller lesions according to recent guidelines in case of single NETs <10 mm with a favorable grading (G1), the decision between endoscopic resection or surveillance should be made on an individual basis (3). In our series, the surveillance strategy was not initially applied to any of the diagnosed gNETs. In fact, a biopsy is required to confirm G1 grade, and this usually results in the complete resection of small gNETs, which makes up the majority of NETs in our patients. Furthermore, our series also includes patients who were enrolled before the publication of more recent guidelines, at a time when resection was considered the safest approach.

Recent evidence suggests that clinical and pathological factors (such as a history of recurrent gNETs, patient age and comorbidities, the presence of a G2-G3 tumor, and the tumor site) should be considered when determining the most appropriate management strategy (19). Nevertheless, endoscopic surveillance alone seems equally acceptable, as evidenced by large retrospective studies that corroborate the generic low risk of tumor progression for type I gNETs <10 mm, while also suggesting a dilation of the endoscopic surveillance schedule (20). It should be noted that despite the paucity of robust data on this matter, it may be reasonable to consider the upfront removal of G2-G3 tumors regardless of their size, given their potential for more biologically aggressive behavior when compared with their G1 counterparts (19).

With regard to preoperative staging, it is undeniable that in the last decades EUS has gained increasing popularity. While past guidelines (17) recognized its potential in locoregional evaluation, they did not provide a cutoff in terms of size or grading when defining its indication. Conversely, more recent guidelines (3) narrowed EUS utilization to lesions >1 cm and to smaller G2 lesions with grading with a high Ki67 proliferation index, even in absence of a defined cutoff value. In our series, preoperative EUS was performed in 13 cases (36.1%), 50% of them being lesions of at least 10 mm of diameter. It is worth noting that our series is retrospective and includes patients who were enrolled prior to any recent guidelines being published. For this reason, even lesions smaller than 10 mm were evaluated using EUS in some cases. In one case, only the EUS evaluation revealed lymph node involvement leading to the patient being indicated surgery according to the mentioned guidelines, whereas in every other case, EUS yielded a negative result and consequently did not change the planned management. The limited role of EUS in this series may also be due to the high proportion of lesions measuring less than 10 mm that were evaluated. While indubitably for type I gNETs >10-mm EUS represents a valid tool to assess the lesion depth of invasion and regional lymph node involvement, and consequently confirm the appropriateness of their endoscopic resection (21), data associating EUS utilization to an improved eradication rate are scarce (13). It is indeed to be remembered that from an economic standpoint, its usage is also associated with significant higher costs and contributes to increased waiting times. Moreover, past studies reported how EUS is characterized by a lower accuracy in the staging of submucosal lesions when compared with the histologic examination of the resected specimen (22). All considered, additional data may be needed in order to better understand the impact of EUS in the management of type I gNETs (19).

In both guidelines, the preferred techniques for the resection were identified as endoscopic EMR or ESD; moreover, the updated 2023 guidelines included endoscopic full thickness resection (FTR) as a possible treatment in case of R1 resection after ESD with a step-up approach (3). Hot-EMR was the most commonly used technique in our cohort (52.9%). A single patient with a 14-mm G1 lesion underwent ESD as a first-line treatment. No cases of endoscopic FTR were reported. Other NETs were removed using biopsy forceps (2 cases, 11.7%) and cold snare polypectomy (2 lesions, 11.7%). The utilization of non-guideline-approved resection techniques is however not uncommon in current literature. This is due to the fact that, as a consequence of the frequent detection of gNETS at very small sizes (<5 mm), biopsy sampling may result in complete resection (23). Nevertheless, previous research has demonstrated that excisional biopsy and cold-snare polypectomy are linked to a high recurrence rate (up to 60%), indicating that these techniques should not be employed with a therapeutic intent (24).

In our cohort, the evaluation of margins in resected specimens was feasible in a limited number of cases (25%). Only one case of R1 resection was documented following a hot-EMR resection of a 13-mm lesion that subsequently exhibited recurrence, but with no impact on overall survival. Data from current literature on the risk of recurrence after R1 endoscopic resection are limited. While a recent review by Esposito et al. (25) reported a cumulative risk of R1 resection after guideline-approved techniques as high as 36% (7.1%-17% and 2.6%-5% for EMR and ESD, respectively), no cases of tumor-related deaths were reported. Moreover, a recent retrospective study demonstrated that in patients with gastric, duodenal, or rectal NETs, G2 grading was the only feature significantly associated with disease recurrence, whereas recurrence after R1 endoscopic resection occurred in a relatively low proportion of patients (8.6%), and even in those cases, it did not impact overall survival (26).

It Is noteworthy that, irrespective of the margin status, in the current series a high recurrence rate (27.7%) has been reported. Data regarding cumulative recurrence rate after endoscopic resection in current literature are quite heterogeneous. Esposito et al. reported a recurrence rate as high as 56.9% even after R0 resection (18), which corroborates the absence of a direct correlation between recurrence/disease progression and the risk of R1 resection. Comparable figures (63.3%) have been documented in earlier studies (24), whereas a recent study (27) did not report any case of recurrence after endoscopic resection.

In fact, the appearance of a new NET in follow-up of a resected gNET type 1 may be interpreted more as a metachronous multifocal disease in a mucosa at risk than as a true recurrence of the previous lesion. In this context, it is important to acknowledge that the evaluation of recurrence in this context is technically complex due to the frequent coexistence of multiple concomitant gNETs, which can be characterized by small size or even be intramucosal NETs, and metachronous lesions that continuously develop in the context of dyschromic metaplastic mucosa, which is pre-cancerous in nature (24). Even guideline-approved resection techniques such as EMR could be burdened by a recurrence rate as high as 18.2% (8). Technical expertise in EMR may also play a role, since more recent works observed no recurrence after 5 years of follow up (28). However, despite the relatively high incidence of recurrence following endoscopic resection, the impact of such recurrences on overall patient survival remains uncertain (13).

In this context, as a future perspective, the use of artificial intelligence (AI) may be beneficial. The implementation of machine learning systems has indeed the potential to effectively assist the endoscopist in identifying high-risk gastric lesions that may be indicative of NETs, thereby enabling upfront endoscopic resection. Furthermore, the utilization of AI in endoscopic surveillance following the resection of gNETs could facilitate a more accurate monitoring of recurrence in patients with high risk factors, thus allowing a less intensive endoscopic surveillance regimen whenever appropriate.

It should be noted that the present study is subject to certain limitations, including its limited sample size and follow-up period together with its retrospective nature. However, the present paper offers a valuable real-life experience from a NET tertiary center with considerable expertise in endoscopic resection techniques and the availability of multidisciplinary approach.

## Conclusions

In conclusion, data from our series align with current literature showing an indolent behavior for type I gNETs. While being strongly recommended by current guidelines, preoperative EUS staging led to a significant change in the therapeutic management in one case only in current series, which highlights the need of dedicated studies in order to identify predictive factors to properly stratify risk and consequently plan the diagnostic and therapeutic management of these neoplasms. As a future perspective, AI may be helpful in early diagnosis and surveillance of high-risk patients; however, further studies are needed.

## Data Availability

The raw data supporting the conclusions of this article will be made available by the authors, without undue reservation.
